# Supplementary material for: Are carbon emissions trading and green financial instruments synergistic? -Comprehensive quantitative research based on content analysis
Source: PLoS One. 2024 Mar 7;19(3):e0298601. doi: 10.1371/journal.pone.0298601 (PMC10919601; doi:10.1371/journal.pone.0298601)
Supplement: S1 Data — (ZIP) [file pone.0298601.s001.zip › Data/地级市Z_F工作报告.zip!/║╙─╧/░▓╤⌠╩╨/└┤╫╘╣1⁄2╓┌║┼í╛┬φ┐╦╩2╛▌═°í┐_2018.docx]

各位代表：

    现在，我代表市人民政府，向大会报告工作，请予审议，并请各位政协委员和其他列席人士提出意见。

    一、本届政府工作回顾

     2014年以来，面对复杂严峻的形势和艰巨繁重的改革发展任务，在省委、省政府和市委的正确领导下，全市人民以习近平新时代中国特色社会主义思想为指导，牢记习近平总书记调研指导河南工作时的殷殷嘱托，主动适应经济发展新常态，坚持稳中求进工作总基调，统筹推进稳增长、调结构、促改革、惠民生、防风险各项工作，全市经济社会发展稳中有进、稳中提质、稳步向好，助力中原更加出彩、决胜全面建成小康社会、实现“一个重返、六个重大”迈出坚实步伐。

    ——经济实力持续壮大。2017年，全市生产总值2017.8亿元，年均增长7.7%；人均生产总值5万元，较2013年末增加1.7万元。固定资产投资2084亿元，是2013年末的1.6倍。社会消费品零售总额735.2亿元，是2013年末的1.4倍。

    ——质量效益持续提升。一般公共预算收入达到118.9亿元，年均增长8.5%；支出258.3亿元，年均增长9.6%；税收占比71.8%。规模以上工业利润总额130亿元。亏损企业亏损额下降10%。万元生产总值能耗下降25.4%。

    ——经济结构持续优化。三次产业比为7∶49.8∶43.2，服务业占比较2013年末提高12.1个百分点，对经济增长贡献率达到55.7%。工业结构深度调整，新兴产业快速发展，高新技术产业增加值年均增长15.7%。农业综合效益明显提升，农业产业化集群收入突破300亿元。常住人口城镇化率达到55.3%。

    ——生态环境持续改善。生态文明建设体制机制逐步完善，污染防治攻坚强力推进。主要污染物排放总量明显下降，PM10和PM2.5浓度分别降至139微克/立方米、82微克/立方米，优良天数增加到185天。水污染防治取得明显成效。绿色发展理念和环境保护意识深入人心。

    ——民生福祉持续增多。财政民生支出累计701.9亿元，占一般公共预算支出的76.8%。居民人均可支配收入22972元，年均增长9%。社会事业全面发展，人民群众有了更多获得感。

    我们主要做了以下工作：

    （一）主动适应经济发展新常态，经济运行稳中有进。面对“三期叠加”“四降一升”的复杂局面，加强精准施策，促进消费升级、有效投资良性互动。扩大有效需求。稳步实施扩投资计划，持续强化项目带动，省、市重点项目累计完成投资2300亿元。西北绕城高速公路、引岳入安等项目进展顺利。第八水厂建成投用。环城路网17个“上跨下穿”重大桥梁工程加快建设。实施“十大扩消费行动”。进出口总额44.1亿美元。电商交易总额280亿元。接待国内外游客4743万人次，旅游总收入436亿元，分别较2013年末增长73%和139%。服务实体经济。狠抓“政策落实进万企”、稳工业“四项对接”活动。设立产业发展基金21支，总规模809.6亿元。拓宽企业融资渠道，累计直接融资659.9亿元。阶段性降低“五险一金”缴费比例，取消或停征、免征政府性基金和行政事业性收费80余项，为企业减负1.5亿元。各类交易市场挂牌企业97家。金融机构存贷款余额分别达到2473亿元和1301亿元。调整完善土地利用总体规划，建立土地大储备机制。推进职教攻坚、全民技能振兴，累计培训98.1万人次，培养高技能人才2.7万人。防范化解风险。强力处置非法集资，贞元、超越等案件取得重要进展。专项治理“半拉子”工程，复工项目46个，盘活资产280亿元。全省首创债权人委员会制度，组建中小企业担保集团、资产管理平台公司，设立企业应急周转金，有效防范企业担保链、资金链风险。推进市场化“债转股”。严格监管、严控规模，政府债务保持在合理区间。战胜“7·19”特大暴雨洪涝灾害，灾后重建任务全面完成。

    （二）牢牢把握转型升级主方向，结构调整步伐加快。坚持以供给侧结构性改革为主线，制定实施“1+6”转型发展攻坚政策体系，推动产业结构优化、生产效率提升、发展动能转换。工业结构不断优化。培育新兴产业，发展高技术、高成长、高利税产业。引进一批新能源汽车及零部件项目，合众德力、比亚迪、福龙马等项目加快实施。建成机器人小镇标准化厂房25万平方米。航空运动城加快建设，通航制造中心建成投用，金宇航空、飞鹰航空等项目签约落地，壮龙、天翼等无人机项目建成投产。改造提升传统产业，实施技改提升工程，推进工业深度治理、提标改造，钢铁、煤化工等传统行业重点企业扭亏为盈。安钢冷轧全线贯通，家电板下线。焦化行业衍生产品有60多种。大力淘汰落后产能。压减煤炭产能40万吨。取缔“散乱污”企业3017家。现代服务业快速发展。推动健康养老、现代物流、文化旅游等产业转型增效。加快旅游资源整合、管理机制调整，组建文旅集团。开通旅游专线10条。成功入围全国首批十大研学旅游目的地城市。红旗渠·太行大峡谷晋升5A级景区。羑里城获批海峡两岸交流基地。百亩以上物流园区达到8个。引进金融机构33家。引进知名电商平台11家，培育本土电商平台9家。大数据云计算中心建成投用。“互联网+”39个专项行动稳步实施。农业供给侧结构性改革深入推进。实施“五大工程”，推进“四优四化”。优质专用小麦、优质花生种植面积分别达到70万亩、48万亩。玉米种植面积调减18%。建成高标准粮田193万亩、生态循环农业45万亩。高效设施农业达到27万亩。培育省级以上畜禽养殖标准化示范场27个、规模以上农业龙头企业408家。发展现代农业园区165个。培育家庭农场1032家、农民专业合作社4181家、“三品一标”214个。农村土地规模经营比重达到50%。

    （三）全力打好污染防治攻坚战，环境治理深入推进。贯彻落实习近平生态文明思想，树牢“绿水青山就是金山银山”的理念，建立完善污染治理目标体系、工作体系、评价体系、考核体系，以前所未有的决心和力度治污染保生态。铁腕治理大气污染。建立“1+6+7”攻坚体系，实施14个专项行动，健全县、乡、村、组四级环保网格，“三治本、三治标”措施落地见效。工业提标改造项目完成272个。规模以上工业煤炭消费总量压减664万吨。完成“双替代”25.2万户。城区集中供热率从65.6%提高到82.8%。淘汰黄标车、老旧车20334辆。全面供应国六标准汽柴油。施工工地落实“八个百分百”抑尘措施。持续开展城乡清洁行动。严格“以克论净”考核，干线公路和城区主次干道实施全时段机械化保洁。城区每平方公里月均降尘量下降8.8吨，降幅42.5%。落实“四个最严”要求，秋冬季重污染天数大幅减少，在“2+26”城市考核中被评为优秀档次。成功入选全国清洁取暖试点城市。市环保局被评为全国环境保护系统先进集体。加强水和土壤污染防治。制定水污染防治“1+2+6”政策措施。河长制挂牌明责。划定畜禽水产养殖禁养限养区，搬迁清理养殖场134家。大力开展城区黑臭水体治理，封堵城市河流排污口3000余个。“两库一泉”综合整治成效显著。水污染防治在2017年全省考核中获得优秀。制订土壤污染防治攻坚“1+6+9”工作方案，启动土壤污染修复和治理。推进生态建设。开展国家森林城市创建，实施“双环六廊”工程，营造林51.6万亩，森林覆盖率24%。3个国家湿地公园建设试点成效初显。实施水利项目122个，引红入洹、引漳入羑等工程建成投用，东湖闸、西湖闸具备蓄水条件。实行排污权有偿交易制度，推进污染防治政府立法，自然资源资产管理保护显著加强。

    （四）坚持改革开放创新，发展活力不断增强。破除体制机制障碍，干成了一批多年想办而没有办成的事。全面深化改革。相继启动改革631项，完成505项。强力推进国企改革攻坚，省属企业“四供一业”剥离任务提前完成，“60+3+4”改革全面完成，荣获全省国企改革攻坚战突出贡献单位。实施部分区域管理范围和管理体制调整，安阳县与示范区、文峰区与高新区实现套合。市级行政审批事项削减51%，非行政许可审批全面取消。清理中介服务66项，取消无谓证明230项，政府系统权责清单实现全覆盖。电子政务服务平台、政务数据共享交换平台建成投用。公共资源交易市县一体化平台在全省率先上线。汤阴县成为国家级基层政务公开标准化规范化试点。“三十五证合一”全面推开。登记市场主体11万户，年均增长24.2%。全面实施“营改增”。稳步推进公立医院改革。深化城市管理体制改革。基本完成农信社改制。优化调整55家事业单位机构编制。公务用车、县级以下公务员职级并行、交通运输行政执法等改革全面完成。科技、文化、价格、电力、供销社等改革顺利推进。推动全面开放。深度对接融入京津冀协同发展和河南省“三区一群”国家战略。安阳保税仓库、豫北跨境电商产业园投入运行。建立招商引资考核体系、项目落地机制，引进10亿元以上项目141个，开工108个；引进省外资金2175亿元，年均增长10.2%；实际利用外资18.7亿美元，年均增长11.4%。成功举办航空运动文化旅游节、中国（安阳）国际汉字大会等重大活动。大力推进创新。制定实施大众创业万众创新系列政策措施，积极培育创新引领型企业、人才、平台和机构。国家级高新技术企业达到62家。培育科技型中小企业431家。建设院士工作站8家、省级工程技术研究中心28家、重点实验室3家。获得国家科技进步二等奖3项。申报专利2611件。培育国家驰名商标4个。

    （五）夯实基础支撑，城乡面貌明显改善。坚持以人为核心推进新型城镇化，促进城乡融合协调发展，全面实施居住证制度，城镇基本公共服务实现常住人口全覆盖。坚持规划先行。城市总体规划获得国务院批复，国家历史文化名城、区域性中心城市定位更加明确。编制完成总体城市设计，调整优化33个专项规划。安阳城市总体规划、古城保护整治复兴规划获得全省优秀城乡设计规划一等奖。开展文明城市创建。突出以绿荫城、以水润城、以文化城、以业兴城，深入推进百城建设提质工程。实施园林绿化项目293个，新建公园、游园60个，新增城市绿地394.5万平方米。扎实推进古城保护整治复兴工程。完善提升城区路网，新建改造道路128公里。打通文昌大道等8条断头路。人民大道西段快车道建成通车。城区27个主要路口完成分流改建。开工建设综合管廊5个。新建改造污水处理厂2座。新增新能源公交车799台，174组智能候车亭、122个公交港湾建成投用，成功创建全省公交优先示范城市。共享单车、公共自行车突破1万辆。建立健全网格化管理体系。持续开展门头牌匾、违规广告、交通秩序、店外经营等专项治理。拆除违法建设677万平方米。成功入围全国文明城市提名城市。市城市管理局荣获全国住建系统先进集体。推进综合交通运输体系建设。铁路、公路、民航、邮政等运输方式深度融合。安阳机场、绕城高速、安内快速通道等加快推进。安姚路、大白线竣工通车。促进乡村改革发展。综合整治农村人居环境，持续完善农村基础设施。农村土地承包经营权确权登记颁证完成。“两权”抵押贷款试点扎实推进。完成永久基本农田划定。解决88.5万农村居民饮水安全问题。1808个行政村完成电网改造。新改建农村公路1544公里。2204个行政村开通电商服务站。建成基层综合文化服务中心1445个。省级以上传统村落达到45个。启动农村“厕所革命”。美丽乡村建设有序推进。

    （六）全力保障和改善民生，社会大局保持稳定。坚持把人民对美好生活的向往作为奋斗目标，保基本，兜底线，建机制，解难题，持续办好重点民生实事。全面推进脱贫攻坚。制定出台“五方案、五办法、五专项”政策措施，建立政策体系、标准体系、责任体系，开展驻村帮扶，推动精准扶贫、精准脱贫。财政投入专项扶贫资金8.6亿元。金融机构发放扶贫贷款30.4亿元。建成产业扶贫基地358个、光伏扶贫电站581个。贫困家庭劳动力转移就业1万人。所有贫困村实现通硬化路、通邮和饮水安全。创新构建健康扶贫“五重医疗保障”，在全省率先建立县级农村贫困人口政府医疗保障专项救助基金。全面推开“六改一增送温暖”活动。243个贫困村实现“七通七有”。9037人完成易地搬迁。11.26万贫困群众实现高质量脱贫。贫困发生率由2013年末的5.63%下降到0.84%。稳定扩大就业。城镇新增就业33万人。新增农村劳动力转移就业14.3万人。发放创业担保贷款26.54亿元。扶持3.9万人次创业，带动就业13.7万人次。强化社会保障。社会保险基本实现法定人群全覆盖。企业退休人员基本养老金实现13连增，城乡居民月基础养老金由60元增加到80元。医疗保险制度全覆盖，异地就医实现直接结算。城乡低保月人均财政补助标准分别提高到250元和142元。农村特困人员集中供养、分散供养标准分别提高到每年5000元和3500元。城乡养老服务设施覆盖率分别达到74%和68%。困难残疾人生活补贴和重度残疾人护理补贴制度全面建立。累计分配保障房15717套，改造农村危房1.2万户。发展公共事业。在全省率先实现县域义务教育均衡发展目标。实施“全面改薄”项目1081个。落实扩充城镇义务教育资源五年规划，新增城镇中小学校学位7.4万个。市一中新校区开工，市六十九中投用。新建、改扩建幼儿园1095所。高等教育、职业教育、民办教育快速发展。加快健康安阳建设。市人民医院新院区建成投用。县级疾控中心、乡镇卫生院等10个基层医疗项目主体完工。全面二孩政策平稳实施。基层公共文化服务不断完善。文物保护持续加强。全民健身运动广泛开展，竞技体育水平不断提升。市儿童福利院新院建成投用。老龄、妇女儿童、红十字、社会福利、慈善和关心下一代工作取得新成绩。创新社会治理。社区管理服务创新示范社区达到200个。安全生产形势持续好转。创建国家食品安全示范城市，建成食用农产品质量检测室32个。依法做好信访工作。构建立体化社会治安防控体系，社会大局和谐稳定。

    贯彻落实全面从严治党部署，加强党风廉政建设和反腐败斗争。深入开展党的群众路线教育实践活动、“三严三实”专题教育、“两学一做”学习教育。自觉接受人大监督、政协民主监督和社会监督，认真办理人大代表建议和政协提案，支持民主党派、工商联、无党派人士参政议政。依宪依法履行政府职能，强化行政监察和审计监督，政府自身建设和治理能力不断提升。国防动员建设深入发展，民兵调整改革任务圆满完成。军民融合持续推进。第六次荣获全国双拥模范城，海军安阳舰顺利入列。老区建设不断加强。统计、档案、史志、气象、地震、民族宗教、外事侨务、发展研究、援疆等工作实现新发展。

    各位代表! 这些成绩的取得，是在市委的正确领导下，全市人民认真贯彻以习近平同志为核心的党中央决策部署，团结拼搏、持续奋斗的结果。特别是党的十八大以来，以习近平同志为核心的党中央举旗定向、把舵领航，推动党和国家事业取得历史性成就、发生历史性变革、实现历史性跨越，在统揽“四个伟大”实践基础上产生形成习近平新时代中国特色社会主义思想，为我们战胜挑战、推动发展指明了前进方向，是我们取得各项成绩的根本所在。在此，我代表市人民政府，向奋斗在全市各条战线上的广大工人、农民、知识分子，向驻安解放军指战员、武警官兵、公安民警及社会各界人士，致以崇高的敬意！向关心支持安阳发展的港澳台同胞、海外侨胞和国际友人，表示诚挚的感谢！

    回顾过去的工作，我们也清醒认识到，全市发展还面临不少困难和问题。一是结构矛盾突出。产业结构不合理，冶金建材、煤化工等传统产业占比重，产品竞争力不强。新兴产业正在培育，尚未形成强大支撑。动能转换困难，要素配置市场化程度低，制约明显加剧，高素质人才短缺，创新能力和科技成果转化能力弱，一些企业特别是中小企业经营困难，民间投资增势减弱。二是环境治理压力大。高污染高耗能高排放企业数量较多、布局不优，煤炭消费总量多，汽车运输占比高，污染物排量大，环境容量严重超载，资源环境对发展的约束日益明显。三是风险隐患增多。企业风险、金融风险和政府债务风险都不容忽视。四是民生欠账较多。脱贫攻坚任务艰巨，在环境卫生、食品药品安全、住房、教育、医疗、就业、养老等方面还存在短板，群众还有不满意的地方。五是政府职能转变还不到位。一些政府工作人员能力素质不适应新时代要求，服务意识、法治意识不强，工作作风不实，形式主义、官僚主义不同程度存在。一些领域不正之风和腐败问题不容忽视。对此，我们一定勇于担当，切实解决，决不辜负人民的重托。

    二、今后五年的目标任务

    党的十九大确定了新的宏伟目标。今后五年，是决胜全面建成小康社会、开启全面建设社会主义现代化新征程的关键时期。市委十一届六次全会提出了推动经济发展高质量和党的建设高质量的明确要求，绘制了助力中原更加出彩的“路线图”“任务书”。我们要看到，落实好、推进好高质量发展，必须跨越两大关口，即必须跨越非常规的经济发展现阶段特有关口，打好防范化解重大风险、精准脱贫、污染防治三大攻坚战；必须跨越常规性的长期关口，打好产业结构优化升级、创新驱动发展、基础能力建设和新型城镇化“四张牌”，破除发展瓶颈，解决深层次矛盾和问题。还要看到，推动高质量发展也面临诸多有利条件，我国经济稳中向好，正处于一个大有可为的历史机遇期；中央出台的一系列宏观政策效应持续释放，有利于我们稳中求进推动发展；市场主体素质提升，不少企业在市场历练中凤凰涅槃，适应性和抗风险能力显著增强；发展环境不断改善，市场活力持续迸发。只要我们转变理念、遵循规律，紧盯目标、锲而不舍，就一定能够开拓高质量发展新境界。

    今后五年的总体要求是：全面贯彻落实党的十九大精神，以习近平新时代中国特色社会主义思想为指导，认真落实习近平总书记调研指导河南工作时的重要讲话精神，坚持稳中求进工作总基调，坚持新发展理念，主动适应社会主要矛盾变化，坚持高质量发展根本要求，坚持供给侧结构性改革主线，坚持转型发展主攻方向，坚持改革开放创新，坚持以人民为中心的发展思想，统筹推进稳增长、促改革、调结构、惠民生、防风险各项工作，着力发挥优势打好“四张牌”，打好三大攻坚战，促进经济社会持续健康发展，决胜全面建成小康社会，开启安阳全面建设社会主义现代化新征程，全力谱写新时代区域性中心强市建设新篇章。

    今后五年经济社会发展的主要目标是：与全国全省同步全面建成小康社会，基本实现“一个重返、六个重大”，乘势而上全面推进新时代社会主义现代化建设。

    ——经济结构明显优化。供给侧结构性改革深入推进，发展质量、效益进一步提升，现代产业体系基本形成，产业高端化、绿色化、智能化、融合化水平显著提高。四个千亿级主导产业主营业务收入达到3000亿元。服务业占国民经济比重在50%以上。

    ——创新水平明显提升。新经济业态快速发展，以企业为主体、市场为导向、产学研深度融合的创新体系全面形成，大众创业万众创新成为引领经济增长新引擎。高新技术产业增加值占规模以上工业比重在35%以上。科技进步贡献率在65%以上。

    ——基础能力明显加强。以机场、铁路、高速公路为骨干，以国省干线公路为基础，以农村公路为补充，国家级地区性综合交通枢纽地位进一步提升。大力发展清洁能源，构建安全绿色、经济高效的现代能源保障体系。推动互联网大规模使用，构建安全高效的信息网络。推进重大水利工程建设，形成布局合理、蓄泄兼筹、多源互补的现代水利系统。

    ——新型城镇化明显加快。常住人口城镇化率达到全国平均水平。城市规划更加科学，建设管理水平明显提升，城市承载能力明显提高，城乡一体化发展能力进一步增强，乡村振兴战略取得阶段性成果。

    ——环境质量明显提高。持续加强生态文明建设。大气污染治理取得重大突破，水环境质量根本改善，土壤环境质量明显好转。单位生产总值能耗下降10%左右。绿色安阳建设取得重大进展。

    ——人民生活明显改善。现行标准下农村贫困人口全部脱贫。教育、文化、卫生、体育等公共服务更加完善，多层次社会保障体系更加健全，基本公共服务均等化水平显著提高。城乡居民收入与地区生产总值同步增长，居民生活条件和生活质量全面改善。治理体系和治理能力现代化水平持续提升，社会大局和谐稳定。

    各位代表！迈步新征程，我们必须落实“四个着力”、打好“四张牌”、县域治理“三起来”、乡镇工作“三结合”的要求，紧跟时代，把准定位，补齐短板，拉高标杆，把该做的事一步一步做实做优，闯新路，塑优势，增实力，奋力在中原更加出彩中走在前、出重彩！

    三、2018年重点工作

    2018年是全面贯彻党的十九大精神的开局之年，是改革开放40周年，是决胜全面建成小康社会、实施“十三五”规划承上启下的关键一年，也是新一届政府的起步之年。今年全市经济社会发展主要预期目标是：生产总值增长7.5%左右，规模以上工业增加值增长7.5%左右，社会消费品零售总额增长11%左右，进出口保持平稳增长，一般公共预算收入增长8%左右，税收占一般公共预算收入70%以上，居民人均可支配收入增长8%以上，城镇新增就业5.9万人，城镇登记失业率控制在4.5%以内，常住人口城镇化率提高1.5个百分点，研究与试验发展经费占生产总值比重进一步提高，居民消费价格涨幅控制在3%左右，单位生产总值能耗、PM10、PM2.5、空气优良天数等节能减排、环境保护指标完成省下达任务。

    重点做好九个方面工作：

    （一）深入推进供给侧结构性改革。全面落实“破”“立”“降”要求，围绕“1+6”转型发展攻坚政策体系，深入实施16个专项行动，加快发展方式转变、经济结构转型、增长动能转换，提高发展质量效益。

    推动产业结构优化升级。培育四个千亿级主导产业。围绕精品钢及深加工产业，着力减量提质、集中布局、创新增效，重点抓好凤宝特钢热扩车桥、晨盛装配式钢结构、复星合力高强钢筋、立德高磁感取向硅钢、神龙腾达彩钢板等项目。围绕新能源汽车及零部件产业，推动示范区（安阳县）、林州市、内黄县错位发展，建设国家新能源汽车轻量化基地，重点抓好合众德力、福龙马、高晶高端铝轮毂等合作项目。围绕高端装备制造产业，重点抓好比亚迪云轨生产基地、中博轨道交通产业园、智能机器人小镇、精密制造产业园、“三新”产业园、致远覆铜板等项目。围绕文化旅游产业，着力强基础、铸品牌、优服务，发展全域旅游、乡村旅游、研学旅游，力争旅游总收入达到480亿元。加快“三大改造”。抓好绿色园区创建，建成绿色示范工厂2家，支持安钢建设“森林工厂”。完成9家企业智能化改造，建设1个智能工厂、4个智能车间。实施215家规模以上企业技术改造。推动林州市装备制造、内黄县陶瓷、殷都区铁合金产业绿色生产，促进北关区和滑县纺织服装做优做强，实现汤阴县食品医药集聚增效，引导殷都区焦化产业和滑县食品加工产业延链提升，鼓励龙安区发展新材料，加快文峰区（高新区）科技研发生产基地建设。严格环保、能耗、安全、质量等标准要求，建立依法依规淘汰落后产能长效机制。发展壮大现代服务业，促进产业融合。培育现代物流、健康养老、教育培训等新的增长点。抓好中国供销?豫北农产品物流园、安西物流园、万庄公铁物流园、内黄果蔬城冷链物流、象道物流园等高成长性服务业项目。深化“互联网+”专项行动，抓好大数据产业园、城市产业云平台等重点项目建设。加快电商平台推广应用，培育省级示范企业10家、市级示范企业50家。

    优化营商环境，推动实体经济做强做优。弘扬企业家精神，构建“亲”“清”新型政商关系。实现企业登记全程电子化，重大项目审批时间再压减一半。推进工业产品生产许可证简化审批程序和委托下放改革，实行“一企一证”。全面实行市场准入负面清单制度。规范涉企收费，降低企业成本，继续执行阶段性降低企业社保缴费率有关政策。落实支持非公有制经济发展政策措施，促进民营企业发展。开展“双百”融资对接行动，年内新增贷款160亿元，直接融资130亿元。新增挂牌企业30家，新引进金融企业6家。

    推动质量提升、质量强市。弘扬工匠精神，实施“六大质量提升工程”，创建全国质量强市示范城市。继续开展增品种、提品质、创品牌专项行动，打造一批先进制造业、现代服务业、特色农业知名品牌。推进市民之家、内黄温室大桃2个国家级标准化示范项目建设。加快国家级铁合金质检中心、中原无人机检测中心建设，推进电主轴、有机产品2个省级质检中心建设。

    （二）扩大有效需求。顺应社会需求新变化，加大基础设施补短板力度，持续释放有效需求，促进经济稳健运行。

    扩大有效投资。围绕产业转型升级、创新驱动发展、新型城镇化提质、基础能力提升、精准脱贫攻坚、环境污染防治6个方面，实施项目1000个左右，完成投资1500亿元，其中237个省市重点项目完成投资740亿元。抓好中央预算内投资项目建设。激发民间投资活力。

    推动消费升级。大力发展消费新业态新模式，培育健康、养老、教育、体育、文化、旅游等消费热点。落实新能源汽车车辆购置税优惠政策。加快龙泉健康养老产业园等项目建设。新建改扩建一批社区日间照料中心。推动网购、快递健康发展。鼓励引导绿色产品消费，大力发展共享经济。

    增强出口拉动。推进安阳铁路口岸、保税物流中心建设，创建国家级跨境电商示范园区。搭建外贸服务平台，深化与河南物资集团合作，推动企业由代理出口向自营出口转变。支持外贸企业发展，推动进出口稳定增长。

    （三）坚决打好三大攻坚战。围绕完成年度攻坚任务，明确各方责任，强化政策保障，确保取得显著成效。

    打好重大风险防范化解攻坚战。稳妥化解企业风险，严防担保链、资金链风险。深入推进市场化“债转股”。防控政府债务风险，开展政府债务审计，化解存量债务，规范政府举债行为。防范化解金融风险，强化小额贷款公司、融资担保公司、典当行、融资租赁公司等金融机构监管，专项整治非法金融活动，严厉打击金融诈骗、网络传销等违法犯罪行为。持续推进非法集资陈案化解，严格管控、严厉打击各类非法集资现象。有效防范社会风险，兜住兜牢公共安全底线。

    打好精准脱贫攻坚战。坚持精准扶贫、精准脱贫基本方略，聚焦特殊困难群体，构建持续增收稳定脱贫长效机制。新建扶贫产业基地65个。创新完善金融扶贫模式。实现所有贫困村通班车。落实低保、扶贫“两线合一”。加强政策支持，统筹做好非贫困村精准扶贫工作。注重扶贫与扶志扶智相结合、救急纾困与内生脱贫相结合，推进产业、教育、健康、交通、水利、电力等领域扶贫。今年再减少农村贫困人口6900人，确保38个贫困村退出，巩固滑县脱贫成果，实现内黄县脱贫摘帽。

    打好污染防治攻坚战。巩固蓝天保卫战成果。坚持科学治污、依法治污、全民治污，突出产业结构、能源结构、运输结构、用地结构调整，深化工业深度治理、“散乱污”企业取缔、冬季清洁取暖等治本之策，严格控尘、控车、控煤、控油、控排、控燃等治标之举，推动绿色发展、绿色生产、绿色建设。引导企业寻标、对标、达标、夺标，抓好23个行业超低排放深度治理。实现“散乱污”企业动态清零。实施禁燃区内涉气企业退城搬迁，推动铸造、铁合金、印染、腐竹加工等行业整合入园。抓好清洁取暖、散煤治理，煤炭消费总量再削减3.2%。实施风电光电双百万工程，发展和推广清洁能源。开展柴油重卡专项治理，继续淘汰老旧车辆。创建城市绿色货运配送示范工程。强化网格化监管和现场临场监管。实施绿色调度。加强重污染天气应急管控。加强水污染治理。强化河长责任，健全巡河制度。深入推进“堵污口、清污泥、治污水、净水质”专项清河行动。全域治理黑臭水体。排查整治明沟暗渠，封堵所有违法违规排污口。推动截污纳管、雨污分流。清理河渠沿岸违法建设。严格畜禽水产禁养限养区管理。防范水环境风险。强化水源地保护。全市河流水质优良比例在50%以上。实施净土行动。完成农用地土壤污染状况详查，编制土壤污染治理与修复规划。加强农业面源污染防治，落实农药化肥零增长计划。规划建设静脉产业园。开工建设垃圾焚烧发电项目。做好生活垃圾分类试点工作。严格固体废物和危险废物处置。推进大宗废物资源化利用、污泥无害化资源化处理。全市规模化养殖场粪污处理设施配套率在82%以上，畜禽粪便综合利用率在68%以上。加强生态修复和保护。创建国家森林城市。开展国土绿化行动，营造林19万亩。启动G341、新东北外环绿化。推进国家湿地公园试点建设。加强省级自然保护区管理。严守生态保护红线。推进西部矿区生态修复。强化自然生态空间用途管制。实施生态补偿制度。完善环境保护考评体系。严格环境执法和问责，严惩环境违法行为。

    （四）持续深化改革开放。以纪念改革开放40周年为契机，持续深化改革，全面扩大开放。

    强化改革推动。深化“放管服”改革。抓好“互联网+政务服务”，全面推进依申请行政权力事项在线办理，深化“一网、一门、一次”改革，打造“一次办妥”政务服务品牌。推行审批服务集中办理。开展相对集中行政许可权改革试点。推进“多证合一”和“证照分离”。全面实施“双随机、一公开”监管。推进公共资源交易平台标准化、智慧化建设。深化国企改革。完成“僵尸企业”处置。剥离央企办社会职能。推进企业产权结构、组织结构、治理结构改革。加强国资国企分类监管。开展混合所有制改革试点工作。推行市场化选聘职业经理人，完善薪酬分配和业绩考核制度。严格土地管理利用，推进“三地一矿”综合改革。抓好农业水价综合改革试点。做好新一轮政府机构改革。深化行政执法、公立医院、医疗保险、生态环保、投融资、财税、教育、文化、盐业等领域改革。

    坚持开放带动。深入对接京津冀协同发展、河南省“三区一群”国家战略，建设中原城市群北部跨区域协同发展示范区。突出四个千亿级主导产业，整合优势资源，集中招商力量，抓好市场招商、专业招商、产业链招商、资本招商，着力引进高技术、高成长、高利税企业，严控高污染、高耗能、高排放项目。推动产业集聚区主导产业精准招商、产品技术工艺创新、落后产能“腾笼换鸟”，培育具有竞争力的产业集群。提高项目履约率、资金到位率、项目开工率，确保进入全省前列。利用省外资金增长5%，实际利用外资增长3%。抓好航空运动文化旅游节、中国（河南）国际投资贸易洽谈会等重大活动签约项目落地。

    （五）提高科技创新能力。实施“四个一批”行动计划，发展一批创新引领型企业，培育一批创新引领型人才，建设一批创新引领型平台，引进一批创新引领型机构，加快建设创新型城市。

    培育创新主体。新增国家级高新技术企业16家以上，新培育“科技小巨人”企业50家，实施对外科技合作项目10个以上。重点推进装备制造、生物医药、环保节能等领域83个科技创新引领项目。高新技术产业增加值占规模以上工业增加值比重在25%以上。科技进步贡献率在55%以上。

    打造创新平台。对接中科院、“双一流”高校等大院名校，争取在安设立分支机构或共建新型高端研发平台。加快推进安阳大学科技园建设。新增市级以上工程技术研究中心、重点实验室10家。新增产业技术创新战略联盟及新型研发机构3家。

    汇聚创新人才。创新人才培养和引进机制，完善吸引、留住、激活人才政策措施。实施“慧聚安阳”计划，培养引进高层次创新创业人才和团队。建设双休双创科技示范园区2个。

    （六）实施乡村振兴战略。全面落实产业兴旺、生态宜居、乡风文明、治理有效、生活富裕总要求，编制乡村振兴战略发展规划纲要和三年行动计划，推动农业强、农村美、农民富。

    深化农业供给侧结构性改革。全面落实粮食安全责任制，稳定提升粮食综合产能。新建高标准粮田7万亩。调优调强种养业结构。优质专用小麦、优质花生、设施瓜菜种植面积分别达到100万亩、50万亩、28万亩。农业产业化集群实现销售收入360亿元。新培育示范家庭农场10家、示范农民专业合作社10家。新建扩建现代农业园区暨都市生态农业园区30个。推进农村一二三产融合，发展农业新业态。

    激发农村活力。深化农村土地制度改革，完善承包地“三权”分置制度，稳步推进农村房屋不动产登记，探索盘活用好闲置农房和宅基地。支持滑县开展全国农民住房财产权抵押贷款试点。深化农村集体产权制度改革。壮大农村集体经济。引导金融机构优化乡村网点布局，全面推开“两权”抵押贷款业务。农业规模化经营率再提高5个百分点，新型农业经营主体达到5300个，新增返乡下乡创业1.1万人。

    推动农村各项事业全面发展。实施文明乡风民风建设工程，建成“美丽乡村?文明家园”示范村773个，创建文明村575个。完善农村医疗、教育、文化等公共服务，改善供水、供电、信息等基础设施。新增农村电商服务站点600个。实施农村人居环境整治三年行动计划，完成196个村环境综合整治，林州市、汤阴县农村垃圾治理通过省达标验收。推进农村“厕所革命”和“四好农村路”建设。加快实施安阳东220千伏输变电工程。强化农村基层组织建设，健全自治、法治、德治相结合的乡村治理体系。

    （七）提升文化旅游水平。推进资源整合，理顺管理体制，开创文化旅游发展新局面。

    夯实旅游基础。加快主要景区、精品线路连接闭合，推进曹操高陵、马氏庄园、殷墟等景区联通道路建设。开通高铁站到红旗渠、太行大峡谷、岳飞庙等重点景区旅游直通车。建设安阳游客集散中心。加强灵泉寺、“两库一泉”保护利用。

    突出品牌效应。着眼建设世界一流国家考古遗址公园，编制殷墟保护利用专项规划，建立集中统一的管理体制机制，实施环境整治、违建拆除、土地流转等专项行动，完成殷墟安防工程，提升殷墟安全管理水平，打造殷墟文化旅游品牌。做好纪念殷墟考古发掘90周年系列活动。加快推进周易文化产业园、曹操高陵保护展示工程等18个文化旅游项目。推动红旗渠列入世界文化遗产预备名录。加强区域旅游规划和投资运营策划。降低重点景区门票价格。积极开展安阳研学旅游年、中欧“一带一路”友谊之旅等活动。推进景区升级改造，A级以上景区突破30个。特色乡村旅游示范点突破20个。

    推进文化建设。加强思想道德建设和群众性精神文明建设，培育践行社会主义核心价值观，传承弘扬红旗渠精神。深入实施“双十”工程，培育壮大新型文化业态。繁荣文艺创作，强化文化市场监管。推进大运河文化带建设。成立市非物质文化遗产保护中心。传承和发扬地域传统文化。加快建设市文体中心和中国文字博物馆续建工程。实施文化惠民工程，实现基层文化中心全覆盖。

    加强文物保护。文物安全是文物保护的红线、底线和生命线。要持续增强文物保护意识，深入开展文物安全隐患排查治理、文化遗址保护区违建拆除、打击文物犯罪等专项行动。全面摸清全市国家和省市级文物保护单位底数，一一建立保护管理责任体系和长效机制，强化人防、技防、物防能力建设。以敬畏之心保护珍贵遗产，传承历史文明，守护民族根魂。

    （八）推进城市建设提质。以创建全国文明城市为抓手，以百城建设提质工程为支撑，打造创业之都、宜居之城、幸福之家。

    深化文明创建。实施园林绿化项目185个，年内完成130个，确保22个50亩以上公园按期推进。创建国家生态园林城市。完善城市水系，改造提升南万金渠、邱家沟，推动水体连通、水质净化。推进洹河两岸景观带建设。全面实施古城保护整治复兴工程。持续开展城市“双修”。完成文峰大道中段、紫薇大道示范街区整治。大力拆除违法建设。推进城乡“创卫”一体化。开展“蛛网清理行动”，推进强弱电入地。加快安彩社区家属院、世纪名城等1173个老旧小区改造提升。加强网格化管理，推进“智慧视频云”和城市智能交通体系建设。

    完善基础设施。推进安阳机场、高铁站东广场、郑济高铁安阳段建设。加快路网连通。西北绕城高速、G341东南西南环城、S301东北外环建成通车，S502安内快速通道完成50%以上。完成黄河东路等92条道路新建改建或提升改造。推进“两场一所”建设。完成东风路、安钢大道等18条道路路灯改造。抓好5个地下综合管廊建设。实现城区地表水供水全覆盖。加快推进城市生活垃圾收运系统升级改造二期、东部南部供热管网、集中供热天然气调峰热源工程。

    优化国土空间格局。启动新一轮城乡总体规划修编。完善综合交通、基础设施、公共服务设施等专项规划，推进“多规合一”。实现中心城区控规全覆盖，划定城市开发边界，严控开发强度。推进“三维”辅助决策系统建设。

    （九）增进人民福祉。践行以人民为中心的发展理念，兜牢民生底线，让人民群众享有更多获得感、幸福感。

    持续增加居民收入。健全收入分配机制。调整社会最低工资标准，提高一线劳动者报酬，增加低收入群体收入，扩大中等收入群体比重，确保居民收入与经济同步增长。

    稳定和扩大就业创业。深入实施职业教育攻坚和全民技能振兴工程，建设大学生就业见习基地、创业孵化园区和新型众创空间。促进高校毕业生多渠道就业。新增农村劳动力转移就业2.2万人。依法治理拖欠农民工工资问题。做好退役军人安置、管理和保障工作。

    完善社会保障体系。做好社会保险扩面工作。调整企业和机关事业单位退休人员基本养老金。推进医保支付方式改革。落实困难残疾人生活补贴和重度残疾人护理补贴。开工建设市残疾人康复中心。加快市社会福利院老年养护楼建设。加强县级社会福利和残疾人康复托养设施建设。

    保障群众住有所居。实施棚改三年行动计划，打破“城中村”自然界限，实行整街区征收改造。加强房地产市场调控，深化住房制度改革，构建多主体供给、多渠道保障、租购并举的住房制度。

    发展人民满意的教育。持续实施第三期学前教育行动计划。推动城乡义务教育一体化发展。支持和规范社会力量办学。加快消除大班额问题。开展校外培训机构专项治理。提升高中阶段教育水平。推动职业教育校企合作、产教融合。促进高等教育内涵式发展。加快市一中新校区、市实验幼儿园新园等项目建设。改扩建农村寄宿制学校16所。

    建设健康安阳。开展区域医疗中心建设三年行动。推进分级诊疗和医联体建设。开工建设市公共卫生服务中心。确保市第五人民医院病房楼主体完工。开工建设内黄县第二人民医院医技综合楼等7个基层医疗项目。确保县级妇幼保健院、乡镇卫生院等11个项目建成投用。

    推进社会治理创新。加强防灾减灾救灾体系建设。严格落实安全生产责任制，强化重点行业重点领域专项治理。加强食品药品监管。创建标准化社区35个。完成村（社区）“两委”换届。做好信访工作。完善社会矛盾排查预警机制。加强困难群体法律援助，建设公共法律服务平台。深化平安安阳建设，健全立体化、信息化、智能化社会治安防控体系。开展扫黑除恶专项斗争。

    支持国防和军队改革，抓好民兵和人民防空建设，深入开展双拥共建，促进军民深度融合。加强老区建设。支持工会、共青团、妇联等人民团体更好发挥桥梁纽带作用。扎实做好老龄和关心下一代工作。加强统计、史志、档案、气象、地震、邮政、发展研究、外事侨务、民族宗教等工作。

    办好重点民生实事：

    1.城乡低保对象月人均财政补助水平分别提高到262元和154元以上，特困人员基本生活标准不低于当地低保标准的1.3倍；城乡居民基础养老金最低标准提高到每人每月98元。

    2.免费开展消化道肿瘤筛查（60岁～69岁居民）；免费开展出生缺陷产前筛查和新生儿疾病筛查；完成适龄妇女两癌筛查5.7万人。为所有乡（镇）卫生院、社区卫生服务中心配备电动喷雾器、红外线体温测定仪，为所有小学、幼儿园配备红外线体温测定仪。

    3.实施“全面改薄”项目148个，扩充城镇义务教育资源规划项目6个，新建改扩建幼儿园56所。

    4.建成基层综合文化服务中心1115个；新建全民健身设施150个。

    5.实施市政道路244项，完成100项，其中背街小巷提升改造58项。建成迎宾公园、CBD公园、龙安公园等8个公园并向市民开放。新增纯电动公交车200台。

    6.新建改扩建城区便民农贸市场、农贸单元、农贸点80个，完成50个；新建改扩建城区便民停车场、洗车点71个，完成40个；新建改建各类公厕379座，改造农村户用卫生厕所30万户左右。

    7.市第四水厂二期、市第六水厂通水试运行。

    8.新开工建设棚户区改造安置房23143套。

    9.全面完成国家和省确定的15个黑臭水体治理。

    10.新建改造农村公路300公里以上。

    各位代表！今年以来，按照市委经济工作会议部署，我们对政府工作紧抓不懈、不等不拖，很多工作已经达成预期。第四季度是全年收官的关键，我们将全力攻坚，大干一百天，向全市人民交上一份满意答卷！

    四、加强政府自身建设

    新时代要有新气象，更要有新作为。必须全面增强履职能力，提升履职水平，深入推进政府职能转变，增强政府公信力和执行力，建设人民满意的服务型政府。

    （一）始终把政治建设放在首位。坚持用习近平新时代中国特色社会主义思想武装头脑，增强“四个意识”，坚定“四个自信”，坚决维护习近平总书记核心地位，坚决维护党中央权威和集中统一领导，在政治立场、政治方向、政治原则、政治道路上同以习近平同志为核心的党中央保持高度一致。扎实推进“两学一做”学习教育常态化制度化，认真开展“不忘初心、牢记使命”主题教育，明大德、守公德、严私德，坚定跟着党中央令旗走，让党的各项方针政策在安阳落地生根！

    （二）推进依宪施政、依法行政。严格遵守宪法法律，坚决依照法定权限、规则和程序，行使权力，履行职责，确保政府活动全面纳入法治轨道，加快建设法治政府。依法接受人大及其常委会法律监督、工作监督，办好人大代表建议、意见，积极回应人大执法检查和专题询问。自觉接受政协和各民主党派、工商联、无党派人士民主监督，办好政协委员提案。依法接受监察委员会监督。自觉接受社会和舆论监督。坚持科学、民主、依法决策，推进政务公开，规范权力运行。政府一切工作都要体现人民意愿，干得好不好，要看实际效果，要由人民评判！

    （三）恪守廉洁从政底线。认真落实全面从严治党主体责任和“一岗双责”，深入推进政府系统党风廉政建设。严格执行中央八项规定实施细则，驰而不息整治“四风”，锲而不舍转变作风。加强审计和巡视巡察结果运用。健全行政权力约束机制，持续强化对政府投资、工程建设招投标等重点领域、关键环节的监督，从源头上预防腐败，以清廉政风赢得人民群众的信任和支持！

    （四）强化为民务实担当。大兴学习之风、调查研究之风、干事创业之风，敢于担当、敢于较真、敢于碰硬。时刻把人民对美好生活的向往作为奋斗目标，凡是群众反映强烈的问题都要严肃认真对待，凡是损害群众利益的行为都要坚决果断纠正。坚持实字当头，干字为先，决不能表态多调门高，决不能行动少落实差，决不能占着位子不干事！改进督查考核问责，完善正向激励和失职追责机制，让改革者轻装上阵、担当者脱颖而出、实干者大显身手，推动各项工作落到实处、取得实效！

    各位代表！新时代是奋斗者的时代，惟其艰难才更需勇毅，惟其笃行才弥足珍贵。让我们在市委的正确领导下，高举习近平新时代中国特色社会主义思想伟大旗帜，不忘初心，牢记使命，以逢山开路的闯劲、抓铁有痕的韧劲、攻坚拔寨的拼劲，为助力中原更加出彩、决胜全面建成小康社会、建设富强民主文明和谐美丽的社会主义现代化新安阳努力奋斗！
